# Annotations

**Published:** 1896-03-07

**Authors:** 


### Annotations.

### One Cause of Hospital Abuse.

We hear a great deal from time to time of the abuse of hospitals by well-to-do people. No doubt the ever-increasing number of persons who seek relief at the hospitals must include a goodly proportion of those who are able to pay a doctor. Hospital abuse is not, however, confined to those who are able to pay. Our voluntary hospitals were established for the benefit of those alone, who whilst able to maintain themselves during health, are unable to provide adequate treatment in the day of sickness. It is an abuse of charity for persons in receipt of poor-law relief, or whose attendance during illness is provided and paid for out of the rates, to obtain relief at the voluntary hospitals. Unfortunately the poor law dispensaries are so conducted at the present time that no self-respecting person will have anything to do with them if he can possibly avoid it. A recent correspondence between an East-end ratepayer and the Local Government Board brings out the fact that "it rests with the guardians, or, in a case of urgency, with the relieving officer, to decide," if a poor person seeking medical relief has a picture on the wall worth a shilling, whether that picture should be sold before the owner or any member of his family is permitted to obtain relief from the poor-law medical officer. It is true that the Local Government Board state that it does not appear to them that the possession of a picture or other article of small value would be regarded as precluding the allowance of relief where otherwise justified by the circumstances. Still, if the evidence given recently at an inquest in East London is to be credited, the parish doctor is not permitted in certain cases to visit a poor person when ill unless or until the picture on the wall has been sold. We venture to think and to say a fact like this should bring home to the hospital authorities, and to people of all classes, the imperative necessity which there is at present for a searching inquiry into the whole organisation under which medical aid is granted and supplied in poor-law cases.

### The "New Child" as "Made in Germany."

Physical training is one of those things that, according to Professor Mosso, of Turin, they do not "do better in Germany." The learned professor lectured before the Roman College of Physical Education the other day, in the presence of the Queen of Italy, and stoutly contended that the German method of training the bodies of the young, which so many were anxious to adopt in Italy, was one of the "worst possible" of all the gymnastic methods employed in European schools. Among other things it was a method which cramped the limbs instead of encouraging and developing their free use. Moreover, it was carried on principally indoors, within the walls and under the roofs of stuffy gymnasia, instead of—as in England—in the open air of the playgrounds and the fields. On the general subject of the modern methods of child education, and more especially as carried on in Germany, Professor Mosso spoke with great decision. He considered that the determination of all classes of school authorities to put all sorts and conditions of school children through the mill of the higher education was so foolish as to practically amount to a "disease." In other words, since it could only be a mental "disease" he was thinking of, he considers that the heads of common education departments in Germany are little better than lunatics. This may appear to some to be strong language; but we cannot help expressing, as we have often expressed before, the idea that in this country also, the determination to educate the children of the industrial poor as if the majority of them were destined ultimately to occupy quite different stations in life is not only a mistake, but a serious injury to the children themselves. In the first place it impairs bodily vigour; and that is a loss which can never afterwards be compensated for. Then, instead of strengthening the brain and increasing the practicality of the children, it often irritates the brain and produces hypersensitiveness, and a lack of courage in facing the rough and tumble of life. Evils of these two classes have already been produced, and their effects may now be seen on a large scale in France. Germany, it appears, is following suit. Let us hope that England will be impressed by the actual results as seen elsewhere, and adopt a more common-sense and practically useful course.

### Mental Defects and Bodily Abnormalities.

The researches in which Dr. Francis Warner has been engaged for some years past in regard to the mental and physical conditions of children are of very great interest, for by them he aims at determining, so far as may be done by statistical methods, the "causes of mental dulness and other defects" often found among the rising generation. The main point brought out is that both defective development and what are spoken of as "nerve signs," *i.e.*, abnormal actions, movements, and tricks in balancing parts of the body, either alone or in combination with other classes of disorder, are much associated with mental dulness. The child which is normal in its nutrition and general development is much more likely to be normal mentally than are children who possess recognisable physical defects and nervous tricks. But there are curious peculiarities in the distribution between the sexes of the deficiencies which are associated with mental dulness. Thus arranging the abnormal conditions which were observed into three main classes, they may be described as cases of defective bodily development, abnormal nervous action, and delicacy of health. Now defective development is more frequent in boys than in girls; and a larger proportion of boys than of girls present one or more class of defect. But when a girl is defective in make, she is more likely to become delicate with brain disorder and mental dulness than the boy. It would seem, in fact, as if boys could, as one might say, "stand" more defective development, without the mental powers being spoiled, than is the case with girls, but that in such cases it is very apt to be associated with nervous disturbances. Perhaps the chief interest of these studies lies in the suggestion, in fact almost the proof, that mental dulness is not a question only of that abstract thing called mind, nor even of brain alone, but is largely associated with bodily defects and with imperfect co-ordination of different portions of the nervous system.

